# Biochemical nature of Russell Bodies

**DOI:** 10.1038/srep12585

**Published:** 2015-07-30

**Authors:** Maria Francesca Mossuto, Diletta Ami, Tiziana Anelli, Claudio Fagioli, Silvia Maria Doglia, Roberto Sitia

**Affiliations:** 1 Unit of Protein Transport and Secretion, Division of Genetics and Cell Biology, IRCCS San Raffaele Scientific Institute, Milan, Italy; 2 Department of Physics, University of Milano-Bicocca; 3 Department of Biotechnology and Biosciences, University of Milano-Bicocca, Piazza della Scienza 2, Milano, 20126, Italy; 4 Vita-Salute San Raffaele University, Milan, Italy

## Abstract

Professional secretory cells produce and release abundant proteins. Particularly in case of mutations and/or insufficient chaperoning, these can aggregate and become toxic within or amongst cells. Immunoglobulins (Ig) are no exception. In the extracellular space, certain Ig-L chains form fibrils causing systemic amyloidosis. On the other hand, Ig variants lacking the first constant domain condense in dilated cisternae of the early secretory compartment, called Russell Bodies (RB), frequently observed in plasma cell dyscrasias, autoimmune diseases and chronic infections. RB biogenesis can be recapitulated in lymphoid and non-lymphoid cells by expressing mutant Ig-μ, providing powerful models to investigate the pathophysiology of endoplasmic reticulum storage disorders. Here we analyze the aggregation propensity and the biochemical features of the intra- and extra-cellular Ig deposits in human cells, revealing β-aggregated features for RB.

To cope with the diversity and unique posttranslational modifications of the secretory proteome, the early secretory pathway (ESP) is rich in chaperones, folding assistants and enzymes that act sequentially as client proteins mature^1^. Owing to this efficient system, aggregation in the ER is not as frequent as in other cellular compartments, despite the complexity and abundance of client proteins^2^,^3^. Proteins with strong tendency to aggregate in the cytosol, such as Hungtintin with expanded poly-glutamine stretches, do not form amyloid fibrils when directed to the secretory compartment^4^. Albeit robust, however, the secretory protein factory is sometimes challenged with insurmountable problems, such as mutants that cannot fold, orphan polypeptides, or clients produced in vast excess. A frequent consequence is the formation of proteinaceous deposits^5^. While in plants these are part of a developmental program, in mammals intraluminal protein deposits often cause diseases. Thus, aberrant proteins that can be neither secreted nor degraded condense in the ESP and cause ER storage disorders (ERSD) with pathogenetic mechanisms that remain largely unclear^6^,^7^,^8^,^9^. Abundant deposits may alter subcellular organization, disturb membrane fluxes and/or trigger different cellular responses.

Given the increasing number of pathological conditions recognised as ERSD, studying the molecular features underlying protein accumulation and condensation in ESP is important to understand which are the pitfalls and solutions that cells deploy to accommodate inconvenient proteins.

Plasma cells housing Ig-containing dilated ESP cisternae (RB in ‘Mott’ cells) are often detected in autoimmune diseases, leukaemias, multiple myelomas, monoclonal gammopathies and chronic infections^10^,^11^,^12^,^13^,^14^,^15^,^16^,^17^,^18^,^19^,^20^,^21^,^22^. The expression of murine Ig-μ chains lacking the CH1 domain can recapitulate RB biogenesis^23^,^24^,^25^. The absence of a functional CH1 domain is also a hallmark of Heavy chain diseases, rare B-cell neoplasms producing an immunoglobulin heavy chain (Ig-H) incapable of binding light chains (Ig-L)^26^. In all Ig classes, the CH1 domain binds the ER chaperone BiP. Ig-L chains displace BiP and assemble into secretion-competent H_2_L_2_ species. In IgM and IgA, these subunits must further polymerize to negotiate secretion^27^. For reasons that remain largely unclear, the absence of a CH1 domain causes an imbalance between the synthesis and the combined rates of secretion and degradation of Ig-H, resulting in their intraluminal accumulation and condensation into detergent insoluble species in ESP. While some of the molecules that regulate RB biogenesis are known (e.g. Ero1α, ERp44, ERGIC53 and PDI; see^24^), information about their biochemical features and the biological consequences of their formation are scarce^5^.

Plasma cells are professional secretors producing large amounts of antibodies^28^. Yet, even in these specialized cells, Ig can aggregate in non-functional species, from crystal bodies to amyloid fibrils^13^, possibly due to their intrinsic variability, high concentration and diverse environments encountered from the ER to the extracellular medium. In AL systemic amyloidoses, Ig-L variants misfold and aggregate into oligomers and ordered amyloid fibrils that affect multiple organs leading to death^29^. Protein aggregates can exhibit different organization levels, from amorphous, to partly or highly ordered (amyloid) structures, intermolecular beta sheets being present in most protein aggregates^30^,^31^.

In this study, we investigated how ordered are Russell Bodies. Our results show that μ∆CH1 form intra- and extra-cellular polymers in many cell types but with different yields, suggesting that its intrinsic propensity to aggregate is tuned by cell specific factors. Moreover, we investigate the aggregation propensity of the μ chain and the biochemical features of μΔCH1 polymers deposited intra- and extra-cellularly.

## Results

### Biochemical properties of RB in lymphoid cells

**Figure 1:**
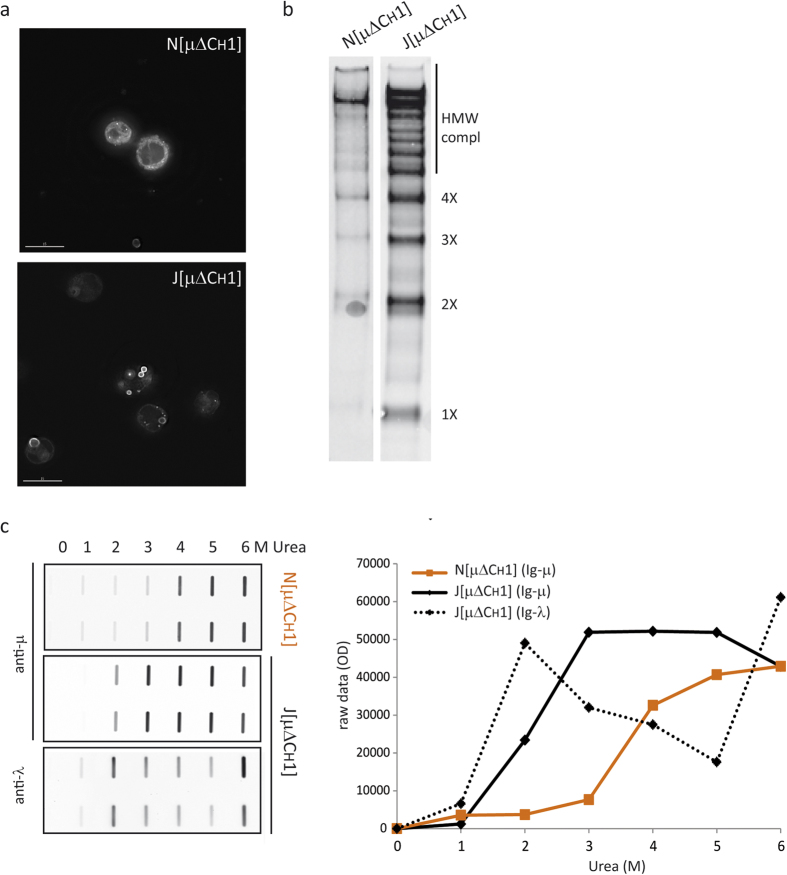
RB formation in lymphoid cells. RB formation in lymphoid cells in the absence (N[μΔCH1]) or presence (J[μΔCH1]) of λ chain. in J[μΔCH1] cells, in the presence of the light chain λ, μΔCH1 forms bigger aggregates (**a** – immunofluorescence with anti-μ antibody) and different assembly intermediates are accumulated in the Tx100 NS material in WB (**b** – Tx100 NS material corresponding to 10^5^ cells loaded under NR conditions on a 2–10% gradient gel) (bar 5µm). **c**) Tx100 NS material from N[μΔCH1] and J[μΔCH1] was subjected to increasing urea concentrations and the fraction of protein released from the pellet was measured by dot blot assay. In the absence of the λ chain, RBs seem more compacted, more difficult to be dissolved by urea. Densitometric quantifications are shown on the right.

**Figure 2:**
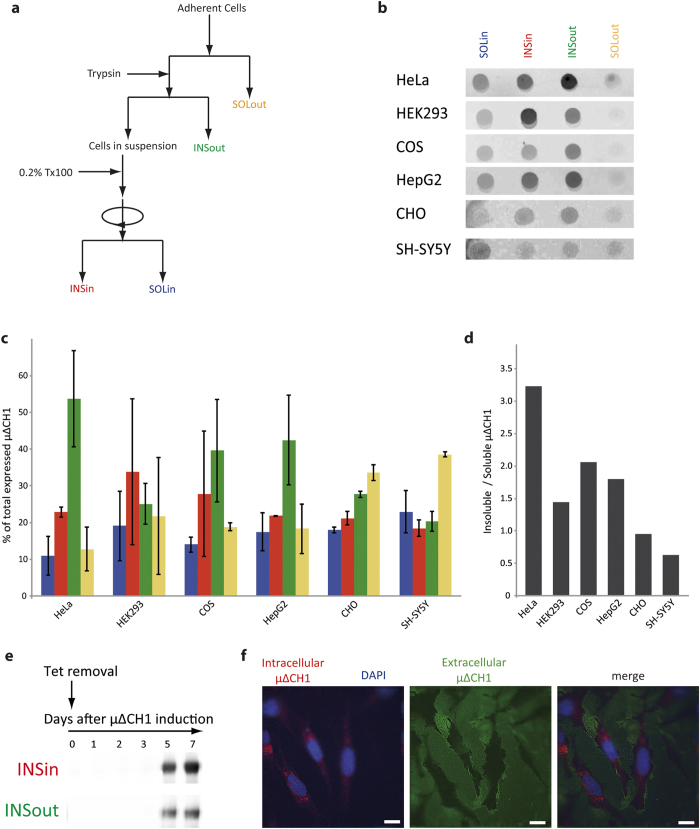
μΔCH1 forms insoluble polymers in different cell lines. (**a-d**) Transiently transfected μΔCH1. **(a**) Intra- and extracellular μΔCH1 species were classified and isolated in four types: intracellular soluble (SOLin-blue), intracellular insoluble (INSin-red), extracellular soluble (SOLout-yellow) and extracellular insoluble (INSout-green). (b-c) Samples corresponding to each fraction were analyzed by dot-blot assay for the presence of μΔCH1. Each point represents the mean ± Standard deviation of two independent experiments. (**d**) The ratio between Insoluble (INSin + INSout) and Soluble (SOLin + SOLout) μΔCH1 varies substantially between different cell lines. (**e-f**) Stable HeLa cell line expressing μΔCH1 under a Tetoff system. (**e**) The INSin and INSout species were isolated at the indicated time points and the presence of μΔCH1 in each fraction was detected by western blot. (**f**) After 7 days of expression induction μΔCH1 accumulates both intracellularly (red) and extracellularly (green), tightly attached to the plastic plate. Scale bar, 5 μm.

In both lymphoid and non-lymphoid cells, RB form when the delicate balance between synthesis, degradation and secretion of Ig-μ chains lacking the first constant domain (μ∆CH1) is misregulated and these aggregation prone mutants condense and accumulate in ESP cisternae^25^. Intriguingly, in the presence of Ig-λ chains, i.e. when expressed in J558L myeloma cells (see Fig. 1a, J[μΔCH1]) or in HeLa co-transfectants^23^ condensation occurs in ribosome studded, rough ER-derived roundish cisternae (rRB). In their absence instead, μ∆CH1 accumulate in smooth vesicular-tubular structures (sRB) containing ERGIC-53 in HeLa^23^. Despite condensation occurs in all cell types tested so far (see Fig. 2 below), important differences exist. In murine NSO myeloma cells that lack Ig-λ (Fig. 1a, N[μΔCH1]), RB display an irregular, cuboid appearance with membranes occasionally studded by ribosomes^23^. Unlike non-lymphoid cells, moreover, neither J[μΔCH1] nor N[μΔCH1] myeloma transfectants secrete μ∆CH1 but retain them intracellularly (our unpublished data) likely reflecting more stringent thiol-dependent quality control mechanisms^24^,^32^.

After synthesis, a considerable fraction of μ∆CH1 chains is insoluble in non-ionic detergents, such as Tx100 (INSin, see below) and can be instead solubilized with SDS. These consist mainly of covalently linked polymers, whilst the soluble fraction (Sin) is enriched in μ∆CH1 monomers and dimers^24^. The distribution of disulfide-linked species differs depending also on the presence or absence of Ig-λ. In the experiment shown in Fig. 1, the Tx100 insoluble fractions were dissociated in 2% SDS, and resolved by SDS-PAGE under non-reducing conditions (Fig. 1b). Clearly, the presence of Ig-λ favored the accumulation of detergent insoluble species, which contained also a ladder of covalent complexes migrating with increasingly faster mobility in denaturing gels. These results suggest that assembly with Ig-λ prevents the degradation of non-polymeric μ∆CH1 and favors their condensation into detergent insoluble species.

Urea-driven dissociation is a powerful tool to differentiate also closely related aggregates, as shown for prion strains^33^. To further investigate the biochemical features of the RB formed in the presence or absence of Ig-λ chains, therefore, we incubated NP40 insoluble pellets from J558L and NSO transfectants with increasing concentrations of urea (0 to 6 M) for 1 h at 25 °C. The samples were then centrifuged at 17000 g (a condition that pellets RB and their contents in the absence of denaturants). Densitometric analyses of dot blots were then utilized to estimate the fraction of μ∆CH1 chains that became soluble upon treatment with urea. Clearly, most μ∆CH1 present in Ig-λ containing, roundish and rough RB were fully dissociated by 3 M urea. In contrast, higher concentrations were needed to solubilize the Ig-λ free assemblies contained in NSO (Fig. 1c,d). Thus, different biochemical forces stabilize these two types of aggregates. Interestingly, a considerable fraction of Ig-λ chains were solubilized at 2 M and more dissociated at 6 M urea, suggesting two modes of binding to μ∆CH1 chains. Our previous findings suggested that interactions between the constant domains of Ig-λ whose variable regions (V_λ_) are non-covalently associated to the V_H_ domains of μ∆CH1 chains that are part of distinct polymers mediate condensation^23^. Low urea concentrations likely weaken V_H_-V_λ_ interactions. The initial detachment of Ig-λ, clearly detectable already at 2 M urea, may set in motion the dissociation of the aggregated material.

### μΔCh1 forms intra- and extra-cellular polymers in different cell lines

Next, we expressed μΔCH1 in non-lymphoid cells that can in part secrete mutant chains even upon polymerization-condensation. As a result, μΔCH1 distributed in four species, detergent soluble (SOL) and insoluble (INS) material being found both intracellularly (in) and extracellularly (out). In all lines tested so far, insoluble species were deposited intra- and extra-cellularly, albeit with different yields. We quantified the four species (isolated 48 h after transfection as schematized in Fig. 2a): intracellular soluble (SOLin - blue), intracellular insoluble (INSin - red), extracellular soluble (SOLout - yellow) and extracellular insoluble (INSout – green, Fig. 2b–c). The ratio between total insoluble (INSin + INSout) and soluble (SOLin + SOLout) material varied considerably (Fig. 2d), likely reflecting the quality control systems deployed by the different cell lines and in particular the levels of those factors, such as Ero1 and ERp44, previously shown to affect μΔCH1 deposition^24^. For example, thiol-mediated quality control is inefficient in HeLa cells, probably owing to the low levels of ERp44^24^. As a consequence, μ∆CH1 was not only deposited as INSin in RB, but it was also abundantly secreted as soluble species found in the cellular medium (SOLout).

**Figure 3:**
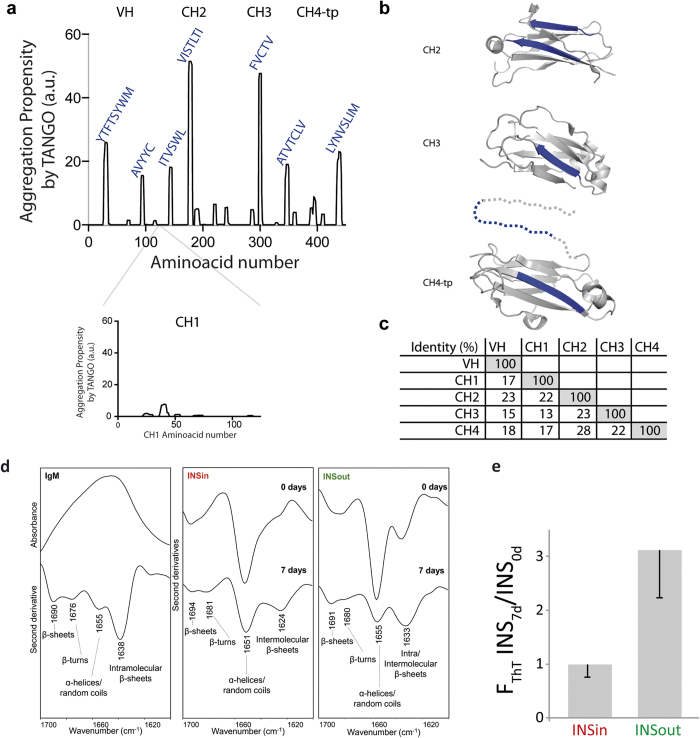
Aggregation properties of μΔCH1. (**a**) The analysis of μΔCH1 sequence with the algorithm TANGO (http://tango.crg.es/)^39^ identifies some aggregation prone sequences, reported on the plot. Aggregation-prone regions are highlighted in blue on the 3D structures of CH2 (PDB 4JVU), CH3 (PDB 4BA8) and CH4 (PDB 4JVW) domains as reported by Muller *et al*.^45^. (**c**) The sequences of the 5 domains of μ chain show a sequence identity <30%. (**d**) ATR-FTIR analysis of purified IgM and isolated INSin, INSout fractions. For purified IgM, the measured spectrum and its second derivative are shown in the amide I region (1700–1600 cm^−1^). The second derivative spectra of INSin and INSout fractions, isolated at 0 and 7 days after tetracycline removal are reported. (**e**) The ratio between ThT fluorescence of INSin and INSout fractions isolated at 0 and 7 days indicates that INSout species bind more tightly ThT. Each point represents the mean ± SEM of 9 (INSin) and 5 (INSout) independent experiments.

To obtain detergent insoluble μΔCH1 in sufficient amounts for biochemical characterization, we took advantage of stable HeLa cells in which a Tet-Off inducible promoter controls expression^23^. Five days after tetracycline removal, abundant detergent-insoluble species deposited both intra- and extra-cellularly. Their deposition increased up to day 7 (Fig. 3e–f). Previous N-glycan analyses suggested that while soluble extracellular μΔCH1 (SOLout) traverse the secretory pathway and are secreted as homodimers, INSout μ∆CH1 polymerize before the transit through the Golgi^24^. In these species, the most carboxy-terminal N glycan (Asn563) remains sensitive to Endo H-because polymerization hinders it to the Golgi enzymes^34^,^35^. For biochemical assays, INSin and INSout species were isolated 7 days after tetracycline removal.

### Aggregogenic features of μ∆CH1 sequence and polymers

Amyloids are protein aggregates characterized by fibrillar morphology, intermolecular cross-β structure and binding of thioflavin T (ThT), a fluorescent dye widely used to estimate the amount of amyloid fibrils in a sample^36^. Almost any protein is able to form amyloid fibrils with these common properties, suggesting that the ability to form amyloid aggregates is an intrinsic property of the polypeptide backbone^37^. However, different sequences vary in their tendency to form aggregates, which can be predicted by several algorithms^38^,^39^,^40^. Amongst the latter, for example, TANGO is able to predict protein sequences prone to form aggregates that are stabilized by β-sheets^39^. Analyzing the sequence of murine Ig-μ chain with TANGO revealed that, of the 5 Ig-μ domains, CH1 has the lowest aggregation propensity (Fig. 3a). During the folding and assembly of IgM antibodies, in fact, while VH and CH2-4 domains acquire their Ig folding independently, CH1 is unique in that it remains unfolded and bound to the ER chaperone BiP until assembly with Ig-L takes place^41^,^42^. The low aggregation propensity of CH1, therefore, has probably evolved to limit aggregation while being in its disordered conformation. Several aggregation-prone regions were instead detected in VH, CH2, CH3 and CH4 domains. When mapped on x-ray structures of CH domains, the aggregation-prone regions identified by TANGO correspond to 3 β-strands and to the conserved tailpiece present in secretory μ chains (μtp). This finding is not surprising, since aggregation propensity has been suggested to be a driving force for macromolecular functional assembly^43^. Nonetheless, stretches with high aggregation propensity could be time bombs in multi-domain proteins such as antibodies. Protein sequences have therefore evolved adopting several strategies to minimize uncontrolled polymerization^44^. To avoid nonspecific aggregation, for example, aggregation-prone interfaces are stabilized in their native conformations by several strategies, such as disulfide bonds and salt bridges, but also by the presence of gatekeeper residues. Indeed, 3 out of 7 aggregation-prone segments identified by TANGO contain a Cys residue involved in an intra-domain disulfide bond^45^. Wright and collaborators^46^, moreover, reported that in polypeptides formed by multiple Ig domains “when the sequence identity falls below a threshold of 30–40% the propensity to coaggregate is negligible”. Indeed, the sequence identity between μ domains is lower than 30%, likely minimizing their propensity to co-aggregate (Fig. 3c).

Ig-μ have therefore some intrinsic propensity to form β-sheet aggregates but the inter-domain identity is just below the threshold of co-aggregation. In our system, however, the absence of the CH1 limits binding of the chaperone BiP and -as a consequence- unleashes the aggregation propensity. Escaping the attentive quality control mechanisms active in the early secretory pathway, μΔCH1 condenses into detergent-insoluble deposits.

To determine if μ∆CH1 aggregates are native-like aggregates condensed in the ESP lumen or are instead stabilized by intermolecular β-sheets we analyzed by ATR-FTIR spectroscopy INSin and INSout fractions isolated 0 days and 7 days after tetracycline removal, *i.e*. in the absence and in the presence of protein aggregates. To this aim we focused on the amide I band region (1700–1600 cm^−1^), due to the C = O stretching of the peptide bond, that is sensitivity to the protein secondary structure and aggregation^31^,^47^. To resolve the overlapping components in the amide I band, we applied second derivative analysis, a widely used resolution enhancement method^48^.

We first measured the FTIR spectrum of isolated IgM (Fig. 3d). The IgM second derivative spectrum is dominated by a main component at ~1638 cm^−1^, due to native β-sheets. Moreover, a minor absorption at ~1655 cm^−1^ is observed, that can be assigned to α-helix and/or random coil structures. In addition, two components at ~1676 cm^−1^ and ~1690 cm^−1^ are found, that are due to β-turns and β-sheets, respectively^47^. As expected, therefore, IgM possess a mainly native β-sheet structure.

At day 0, in the absence of RB, the FTIR spectrum of INSin fraction of HeLa cells is characterized by a main component at ~1651 cm^−1^, assigned to α-helix and/or random coil structures (Fig. 3d). A shoulder around 1624 cm^−1^ is also detected, due to intermolecular β-sheets, characteristic of protein aggregates^31^,^47^. In addition, an absorption at ~1694 cm^−1^, with a shoulder at ~1681 cm^−1^ is detectable, due to β-sheets and β-turns respectively. These spectral features changed after 7 days of intense synthesis and intracellular deposition of μΔCH1. In particular, the α-helix/random coil band decreased in intensity, while the ~1624 cm^−1^ component due to intermolecular β-sheets increased in intensity. These results indicate that the accumulation of INSin polymers induces an easily detectable enrichment in intermolecular β-sheet structures.

As far as INSout fractions are concerned, the spectrum obtained before induction is characterized by two main components one at ~1655 cm^−1^, due to α-helices/random coils, and the other at ~1637 cm^−1^, that can be assigned to intramolecular β-sheets. Upon 7 days of induction, a dramatic reduction of the α-helix/random coil band was observed, accompanied by a downshift of the 1637 cm^−1^ band to ~1633 cm^−1^, indicating that the formation of intermolecular β-sheets was taking place (Fig. 3d). Moreover, a β-sheet component at ~1691 cm^−1^ and a minor absorption around 1680 cm^−1^, assigned to β-turns, were detected.

Since μ∆CH1 is not present in 0 days samples, the FTIR signals detected in these samples are due to the presence of contaminants isolated during the fractionation process. In the case of INSin fraction they correspond mainly to cytoskeleton proteins, such as actin and vimentin, and histones. INSout fractions, instead, are contaminated by other proteins that attach to the plastic plate. Information about μΔCH1 aggregates is therefore provided by the signals changes upon tetracycline induction.

The comparison between the spectral features of INSin and of INSout aggregates (7 days samples), suggests that the two types of aggregates differ in structure. Indeed, the 1624cm^−1^ band found in the INSin spectrum is indicative of more compact structures than those found extracellularly. Here, the presence of a 1633 cm^−1^ band suggests accumulation of looser structures. In particular in the case of INSout aggregates, the broad width of the β-sheet band could be indicative of the formation of a heterogeneous population of β-sheet structures.

Moreover the relative intensity between the band due to α – helix/random coil and that due to intermolecular β sheet suggests that the INSout deposit might contain a higher amount of β sheet structures compared to the INSin one.

To further investigate the conformation of INSin and INSout polymers, we incubated the very same samples analyzed by FTIR with ThT. While ThT fluorescence does not increase significantly for INSin samples between 0 and 7 days, it increases approximately 3 times in the presence of INSout aggregates (Fig. 3e). Despite this small difference, however, both values are negligible when compared to ThT binding to a similar amount of amyloid fibrils.

Unfortunately, these experiments could not be reproduced in plasma cell lines because we were unable to solubilize the NP40 pellets obtained from either NSO or J558L transfectants unless strong denaturants like urea or SDS were added. In these cells, which do not secrete μ∆CH1 chains, also non-physiological Ig polymers such as those lacking CH1, are compacted and hardly manageable for techniques as size fractionation in gradients. These results may correlate with our observation that plasma cell lines do not secrete μ∆CH1 chains, a feature that may reflect their specialization in IgM polymerization, secretion and quality control^32^.

### Biochemistry of μΔCH1 polymers

Surprisingly, INSout aggregates, which are characterized by a looser intermolecular β-sheet structure, bind slightly more ThT than INSin polymers. The signal intensity depends on binding of ThT to specific protein conformations: therefore a low binding can be explained by low amounts or by hampered accessibility of amyloid structures.

**Figure 4:**
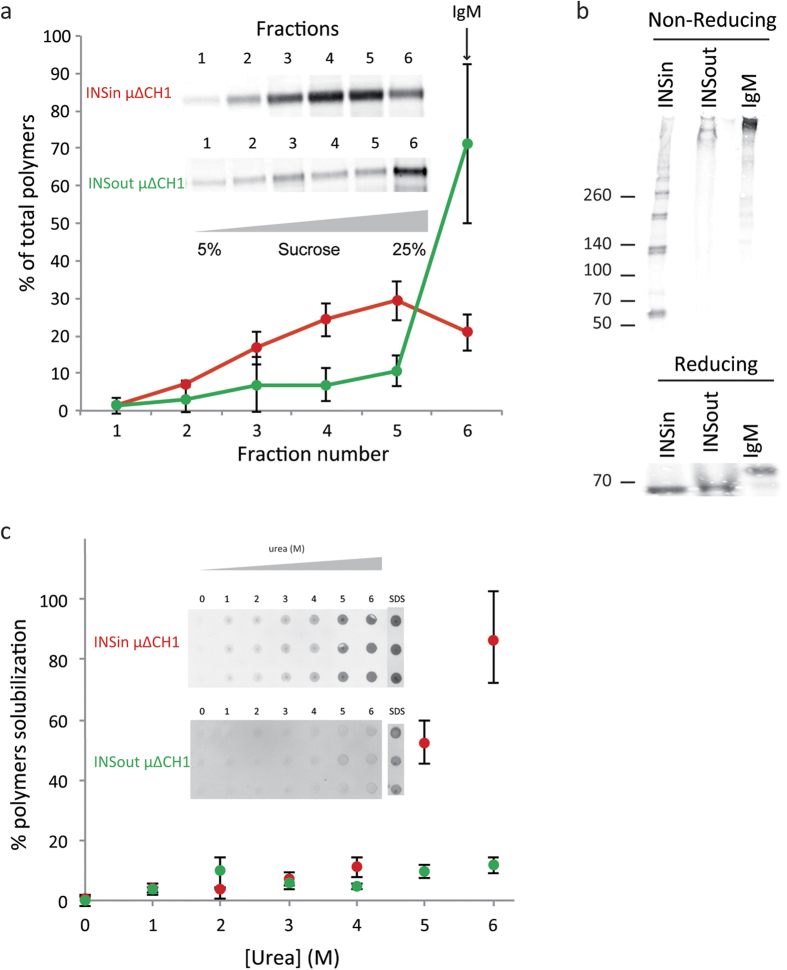
Biochemical features of aggregated μΔCH1. (**a**) Isolated INSin (red) and INSout (green) aggregates were analyzed by SDS-PAGE electrophoresis under not reducing (upper gel) and reducing (lower gel) conditions. Purified murine IgM antibodies were loaded in the same conditions as a control. (**b**) Isolated INSin (red) and INSout (green) aggregates were treated with increased concentrations of urea (0–6 M). The fraction of total protein released from the insoluble pellet and found soluble in the supernatant was measured by dot/blot assay. (**c**) Isolated INSin (red) and INSout (green) aggregates were fractionated by centrifugation on a 5 to 25% sucrose gradient. Fractions of the gradient (lanes 1 to 6) were collected. The amounts of μΔCH1 in each of the fractions of the gradient were quantitated by densitometric analysis of Western blots and plotted as percentages of total μΔCH1. Each point represents the mean ± standard deviation of three independent experiments. Purified murine IgM antibodies were run in a parallel gradient as a sedimentation marker.

To gain more information on the nature of isolated INSin and INSout species, we analyzed their state of polymerization by velocity sedimentation on sucrose gradients. To analyze their size distribution, we centrifuged isolated polymers on a 5 to 25% sucrose gradient at 129,000 g for 3 h and then probed individual gradient fractions by western blotting with anti-μ antibodies, to reveal the presence and state of assembly of μΔCH1. As a comparison, we performed the same sedimentation analyses on IgM antibodies. INSin were found mainly in fractions 4 and 5 while INSout accumulated mainly in fraction 6 (Fig. 4a). Interestingly, the sedimentation pattern of isolated INSin was very similar to the sedimentation pattern of μΔCH1 -containing microsomes obtained from cells 7 days after tetracycline removal (Fig. S1), suggesting that INS isolation did not alter the size distribution of μΔCH1 polymers. These results indicate that INSout μΔCH1 consist of larger species than INSin.

μΔCH1 has 3 cysteine residues (Cys 337, 414 and 575) that can be involved in intermolecular disulfide bonds. To determine if intermolecular disulfide bonds play a role in determining the strength of subunits association we resolved similar amounts of INSin and INSout by SDS-PAGE in the presence or absence of reducing agents (Fig. 4b lower and upper panels, respectively). While under reducing conditions INSin and INSout polymers run as a single band corresponding to the monomeric μΔCH1 chain (~67 kDa), under non-reducing conditions INSin run as a mixture of dimeric to multimeric species, while INSout run as high molecular weight species comparable, if not slower migrating than isolated IgM (>970 kDa). Considering that wt IgM contains covalently bound L chains, absent in these μ∆CH1 preparations, INSout are most likely formed by the covalent cross-linking of μ∆CH1 polymers, or by the insertion of >12 chains in a single polymer.

To determine whether INSin and INSout differed not only in size but also in how “tightly” their subunits were associated, we assessed their susceptibility to dissociation by urea as described in Fig. 1 for RB in lymphoid cells. At all urea concentrations tested, INSout were less efficiently dissociated into soluble forms (Fig. 4c). At 6 M urea, only <10% of the INSout is solubilized compared to 80% of INSin. These results indicate that the two forms of μΔCH1 polymers differ substantially in the strength of subunits association. The alternative possibility is that unfoldase activities co-purify with intracellular RB is unlikely, since mixing the two preparations did not favor dissociation of the extracellular species (data not shown).

## Discussion

The possibility of inducing μΔCH1 condensation in different cell types offers a powerful model to investigate some of the events that are linked to the pathogenesis of ERSD^49^. An important conclusion that stems from these studies is that different cells manage aggregation-prone mutant Ig-μ chains in different ways. Lymphoid cells, characterized by efficient polymerization and quality control machineries^24^,^25^,^49^,^50^, do not secrete mutant μΔCH1 chains and store them intracellularly in compact deposits, that cannot be solubilized without strong denaturants. Non–lymphoid cells, instead, can secrete structured species and allow biochemical analyses also of their intracellular deposits.

In the non-lymphoid cells tested, μ∆CH1 chains distributed in four pools, differing in their detergent solubility and topology. Some cells secreted less than others, and in some insoluble material predominated, providing powerful systems to investigate the causes and consequences of protein condensation in ESP. Clearly, μΔCH1 chains confirmed their intrinsic propensity to condense and aggregate and our analyses revealed novel interesting features of RB, so far considered Ig waste bins of the ER.

Relatively few physicochemical properties of protein sequences - charge, hydrophobicity, patterns of polar and nonpolar residues, and tendency to form secondary structures - can predict their relative propensities to form amyloid fibrils^38^,^39^,^40^. However, protein aggregation is also regulated by environmental factors, such as the pH, redox state and concentration of chaperones and regulatory ions, such as calcium. These features could explain why Huntingtin expressed in the ER does not form amyloid aggregates^4^ and why secretory proteins in general, known to be more aggregation prone than cytosolic proteins^3^, do not accumulate often in ESP as detergent insoluble deposits. As far as RB are concerned, the cell environment plays a central role in regulating their formation^24^ and packing them into ER tubular structures and cisternae where they do not elicit ER stress responses (A.Orsi, R.Sitia, M.Vitale, and E. van Anken, unpublished results). Chaperones are largely excluded from detergent insoluble deposits and are confined to the periphery of RB^25^. Indeed, being stabilized by intermolecular disulfide bonds and β-sheets INSin deposits are quite viscous^49^, perhaps hampering the access to additional chaperones. The propensity to form intermolecular β -sheet mediated aggregates is dictated by the presence of some aggregation prone sequences in VH, CH2, CH3 and CH4 domains. It is interesting to note that another mutant, μ∆CH1Ala565 is even more aggregation prone according to TANGO prediction, and forms aggregates more rapidly in cells (T.Anelli, C.Fagioli and R.Sitia unpublished results).

Some cells secrete part of the RB contents, perhaps as a defense strategy. Intriguingly, however, intra- and extra-cellular aggregates have different biochemical and structural properties (Table 1). INSin polymers are formed by disulfide-linked dimers and higher molecular weight (HMW) species stabilized by tight intermolecular β-sheets. INSout polymers, instead, are formed mainly by HMW species stabilized by more intermolecular disulfide bonds and looser intermolecular β-sheets. Judging from their glycan processing^24^, INSout polymers form before the transGolgi. We can envisage two different scenarios to account for INSout secretion. Transport is restricted to a given type of aggregates, higher molecular weight polymers that elude quality control. Alternatively, INSout are INSin species that undergo a profound structural reorganization during or upon secretion: mechanical forces, dilution upon secretion and interactions with the plastic plate could indeed affect their structure.

Extracellular aggregates are detected also in many α1-antitrypsin deficiency cases^51^. Characterizing the biochemical feature and the secretion mechanisms of insoluble aberrant polymers could allow the identification of new disease marker and possibly therapeutical targets.

The characterization of INSin and INSout reported here extends our understanding of ESP proteostasis. A comparison with other ERSD-proteins (e.g. alpha1-antitrypsin) would be important to discover common and private pathways of aggregation and to determine if general biological responses to ER aggregates exist and if they are tunable and therapeutically targetable.

## Methods

### Cell lines and culture

All cell lines were purchased from American Type Culture Collection (ATCC) and maintained in DMEM containing 2 mM glutamine and 5% FCS (Hela, HEK293, COS) or 10% FCS (HepG2, CHO, SH-SY5Y). The Tetoff inducible HeLa cell line was described in detail previously^23^.

NSO and J558L cells and plasmids encoding mutated Ig-μ chains (μΔCH1) were described previously^23^,^25^.

### Confocal microscopy

After 7 days on induction, HeLa cells grown on cover slips were washed and directly stained with AlexaFluor488 Goat anti Mouse IgM (Life Technologies, Carlsbad, CA, USA) to detect the presence of extracellular μΔCH1. Cells were then fixed in 4% paraformaldehyde: after permeabilization in 0.1% TX100 slides were stained with AlexaFluor546 Goat anti Mouse IgM (Life Technologies, Carlsbad, CA, USA) to detect intracellular material and Hoechst to visualize nuclei.

### Cellular fractionation

After 48 h after transient transfection or the indicated days of tetracycline-dependent induced expression, the medium was removed to isolate the extracellular soluble μΔCH1 fraction (SOLout). Cells were washed and lysed at the concentration of 1 × 10^4^ cells/μl in buffer A (0.2% TX100, 50 mM Tris-HCl pH 7.5), 150 mM NaCl, 5 mM EDTA, 1 mM *N*ethylmaleimide and a cocktail of protease inhibitors (Roche, San Francisco, CA, USA). The TX100-insoluble fraction (INSin) was separated by centrifugation at 3,400 g for 10 minutes, treated with benzonase for 1 h on ice (to remove DNA) and solubilized in lysis buffer B (1% SDS, 50 mM Tris-HCl pH 7.5) for 10 minutes at room temperature, diluted in 50 mM Tris-HCl pH 7.5, 0.2% TX100, to keep the volume of the soluble and insoluble fractions equal, and sonicated for 10 seconds. The extracellular insoluble fraction (INSout), deposited on the plate, was scraped from the plate using a scraper in 0.1 M Tris pH7.4, 1 mM *N*ethylmaleimide and a cocktail of protease inhibitors (Roche, San Francisco, CA, USA).

### Dot-blot assays

Samples corresponding to 10^5^ cells (5 × 10^4^ for SOLout samples) were loaded on a nitrocellulose membrane, blocked with 5% milk, and visualized by dot blot using an AlexaFluor680 Goat anti Mouse IgM (Life Technologies, Carlsbad, CA, USA) and analyzed by Image J software.

### Western blotting

Samples (Tx100 soluble and non-soluble material, treated with benzonase and buffer B) corresponding to 10^5^ cells were loaded on 10% Bis-Tris precast SDS-PAGE gels (Invitrogen, Eugene, Oregon, USA). Gels were transferred to nitrocellulose membranes and decorated with AlexaFluor680 Goat anti Mouse IgM (Life Technologies). WB images were acquired with the fluorescence Scanner Fuji FLA 9000 (FujiFilm Life Science, Tokyo, Japan). To analyze the presence of intermolecular disulfide bonds isolated INSin and INSout fractions were loaded in a 3–8% SDS-PAGE gel (Invitrogen, Eugene, Oregon, USA) in the presence or absence of 300 mM DTT.

### Sequence alignment

Sequence identity was calculated by using ALIGN Query (GENESTREAM SEARCH network server IGH Montpellier, France).

### ThT assays

Tx100 NS material (obtained after cell lysis with buffer A) was analyzed. ThT binding was monitored by exciting samples at 440 nm and recording the emission fluorescence 510 nm in a Viktor III plate reader (Perkin Elmer, Waltham, Massachusetts, USA). For each measurement, 0.85 μl of a 2.5 mM ThT stock solution prepared in 10 mM phosphate buffer (pH 7.0) containing 150 mM NaCl was added to 2 μL aliquots containing the INSin and INSout fractions of 10^6^ cells and adjusted to 100 μL with phosphate buffer.

### Infrared spectroscopy analysis

Fourier Transform infrared (FTIR) absorption spectra were collected in the Attenuated Total Reflection (ATR) mode, using a single reflection diamond element (Golden Gate, Specac Inc, 500 Technology Court, Smyrna, GA, 30082 5211, USA). This approach is widely used for the study of protein aggregates, since it allows the characterization also of insoluble protein assemblies^31^,^52^,^53^. In particular, the sample is deposited on the ATR crystal where it forms a protein film after solvent evaporation. When the IR beam reaches the crystal at the interface with the protein sample, with an angle that corresponds to that of total reflection, an evanescent wave penetrates into the sample where it can be absorbed. The absorbed light can be then detected in the mid infrared range, giving information on the protein secondary structure and aggregation. For our experiments, approximately 5 μL of sample were deposited on the ATR plate and dried at room temperature in order to obtain a homogeneous film^52^,^53^. The ATR-FTIR measurements were performed using the Varian 670-IR spectrometer (Varian Australia Pty Ltd, Mulgrave VIC, Australia) equipped with a nitrogen-cooled Mercury Cadmium Telluride (MCT) detector under the following conditions: 2 cm^−1^ spectral resolution, scan speed of 25 kHz, 512 scan coadditions, and triangular apodization. The measured spectra were smoothed by a binomial function (13 points) and second derivative spectra were obtained by the Savitzky Golay method (3rd grade polynomial, 9 smoothing points)^48^. The derivative spectra have been normalized at the tyrosine peak, at ~1514 cm^−1^, to account for possible differences in the protein content. In order to verify the reproducibility of the spectral data, more than three sample preparations have been analyzed.

### Urea dissociation

Tx100 insoluble fractions obtained from J558L or NSO were incubated with increasing concentrations of urea for 1 hour at RT and centrifuged. Aliquots of the supernatants were analyzed by slot blot assays and the fraction solubilized calculated as the percentage of total μΔCH1 chains solubilized by SDS.

### Sucrose gradient fractionation

Isolated INSin and INSout aggregates were resuspended in Homogenization buffer (0.25 M Sucrose, 10 mM Tris-HCl pH7.4, 10 μM NEM, proteases inhibitors cocktail (Roche, San Francisco, CA, USA) were fractionated by centrifugation on a 5 to 25% sucrose gradient for 3 h at 129,000 × g in a Sw55Ti rotor using a Optima L-90 k Ultracentrifuge (Beckman, West Sacramento, CA, USA). Fractions of the gradient (lanes 1 to 6) were collected. The amount of μΔCH1 in each of the fractions of the gradient was quantitated by densitometric analysis of Western blots. Purified murine IgM antibodies (Life Technologies, Carlsbad, CA, USA) were run in a parallel gradient as a sedimentation marker.

## Additional Information

**How to cite this article**: Francesca Mossuto, M. *et al*. Biochemical nature of Russell Bodies.. *Sci. Rep*. **5**, 12585; doi: 10.1038/srep12585 (2015).

## Supplementary Material

Supplementary Information

## Figures and Tables

**Table 1:** Features of INSin and INSout polymers.

		INSin	INSout
**Site of deposition**		ERGIC compartment	Extracellular compartment
Glycans processed in the Golgi compartment*	**N332-395–402**	no	yes
	**N563**	no	no
Site of aggregation*		ERGIC compartment	before the Trans-Golgi compartment
**Intermolecular β-sheet**		tight	loose
**ThT binding**		low	low
**Intermolecular SS**		Dimers to HMW species	HMW species
**Density**		medium	high

^*^Data adapted from^24^.
