# Supplementary material for: Biochemical nature of Russell Bodies
Source: Sci Rep. 2015 Jul 30;5:12585. doi: 10.1038/srep12585 (PMC4649990; doi:10.1038/srep12585)
Supplement: Supplementary Information [file srep12585-s1.doc]

**Biochemical nature of Russell Bodies**

Maria Francesca Mossuto^1^, Diletta Ami^2,3^, Tiziana Anelli^1, 4^, Claudio Fagioli^1^, Silvia Maria Doglia^2,3^, and Roberto Sitia^1, 4, *^

^1^Division of Genetics and Cell Biology, IRCCS San Raffaele Scientific Institute, 20132 Milan, Italy

^2^Department of Physics, University of Milano-Bicocca,

^3^Department of Biotechnology and Biosciences, University of Milano-Bicocca, Piazza della Scienza 2, Milano, 20126, Italy

^4^University Vita-Salute San Raffaele, Via Olgettina 58, 20132 Milan, Italy

* Please address correspondence to sitia.roberto@hsr.it


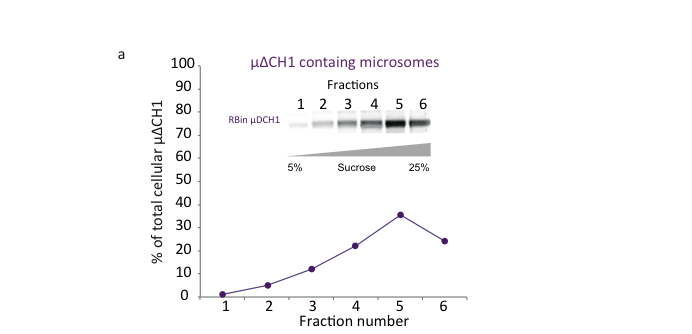


**Fig. S1** Microsomes were isolated from HeLa Tetoff cells after 7 days of µΔCH1 induction. Cells were harvested in Homogenization buffer (0,25M sucrose, 10mM Tris pH 7.4) and homogenized passing 40 times through a 25G needle in Homogenization buffer (0,25 M Sucrose, 10mM Tris-HCl pH7.4, 10µM NEM, proteases inhibitors cocktail (Roche, San Francisco, CA, USA)). The homogenate, freed of nuclei and cell debris by low-speed centrifugation, was then layered on a 5 to 25% sucrose density gradient. After 3 h of centrifugation at 129,000 × g in a Sw55Ti rotor using Optima L-90k Ultracentrifuge (Beckman, West Sacramento, CA, USA), fractions of the gradient (lanes 1 to 6) were collected. The amounts of µΔCH1 in each of the fractions of the gradient were quantitated by densitometric analysis of Western blots and plotted as percentages of total µΔCH1.
